# Synthesis and Structure–Activity Relationships of 4-Morpholino-7,8-Dihydro-5*H*-Thiopyrano[4,3-*d*]pyrimidine Derivatives Bearing Pyrazoline Scaffold

**DOI:** 10.3390/molecules22111870

**Published:** 2017-10-31

**Authors:** Qinqin Wang, Xiaojing Li, Chengyu Sun, Binliang Zhang, Pengwu Zheng, Wufu Zhu, Shan Xu

**Affiliations:** 1 Jiangxi Provincial Key Laboratory of Drug Design and Evaluation, School of Pharmacy, Jiangxi Science & Technology Normal University, Nanchang 330013, China; m15180134379_2@163.com (Q.W.); zbl1045762244@163.com (B.Z.); zhengpw@jxstnu.edu.cn (P.Z.); 2 College of Service, Naval Logistics Academy of PLA, Tianjin 300450, China; 13970892404@163.com; 3 Department of Pharmacy, The Affiliated Hospital of Chongqing Three Gorges Medical College, Wanzhou, Chongqing 404000, China; sunchengyu0902@163.com

**Keywords:** thiopyrano[4,3-*d*]pyrimidine, pyrazoline, synthesis, cytotoxicity activity, PI3Kα kinase

## Abstract

Phosphatidylinositol 3-kinase/Protein kinase B/Mammalian target of rapamycin (PI3K/Akt/mTOR) signaling pathway is abnormally active in the growth and proliferation of cancer cells. The inhibition of PI3K kinase can effectively block the conduction of signaling pathways and is an ideal target for drug design. In this paper; two series of 4-morpholino-7,8-dihydro-5*H*-thiopyrano[4,3-*d*]pyrimidine derivatives bearing pyrazoline moiety (**7a**–**l**; **8a**–**l**) were synthesized; and their cytotoxicity in vitro were evaluated by 3-(4,5-Dimethylthiazol-2-yl)-2,5-diphenyltetrazolium bromide (MTT) method against four human cancer cell lines including A549; PC-3; MCF-7; and HepG2 cell lines. The activity of the most promising compound **8d** against PI3Kα kinase was further evaluated. The results indicated that most of the target compounds showed moderate to excellent cytotoxicity and the most promising compound **8d** showed excellent cytotoxicity against four cancer cell lines with half maximal inhibitory concentration (IC_50_) values of 6.02–10.27 μM. In addition; the compound **8d** was found to have a moderate inhibitory activity in the PI3Kα enzyme assay. What’s more; the compounds of which the substituents of benzene ring at the C-4 position are electron-withdrawing groups such as substituents (Cl; F; Br) have better activity than the compounds containing the electron donating groups (OCH_3_; H). However; the exact action mechanism is not quite clear right now. Further study will be carried out to identify the exact target in near future.

## 1. Introduction

**Figure 1:**
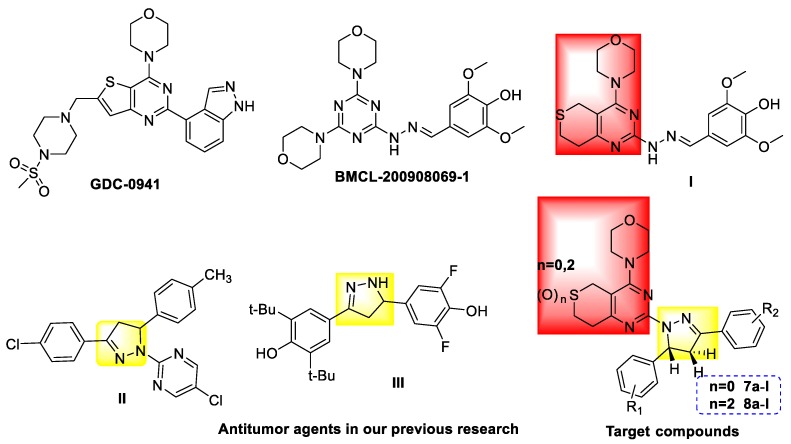
Structures of representative compounds of two kinds of inhibitors and target compounds. Pictilisib dimethanesulfonate (GDC-0941), 4-((2-(4,6-dimorpholino-1,3,5-triazin-2-yl)hydrazono)methyl)-2,6-dimethoxyphenol (BMCL-200908069-1).

Cancer is the main killer of human health, causing high mortality, posing a serious threat to human health. Chemotherapy has been widely applied in various cancer treatments. The toxicity and resistance of traditional chemotherapeutic drugs make it urgent to find new targets and novel drugs for the cancer therapy. The family of lipid kinases termed phosphatidylinositol 3-kinases (PI3Ks) catalyze the phosphorylation of the 3-hydroxyl position of phosphatidylinositides and has been found to play a regulatory role in many cellular processes, including cell growth, proliferation, differentiation, motility, and survival [1]. The PI3K family consists of at least eight proteins that share sequence homology within their kinase domains and yet have distinct substrate specificities and modes of regulation. In addition, PI3K signaling pathway is negatively regulated by phosphatase and tensin homolog deleted on chromosome ten (PTEN), which is one of the most commonly mutated proteins in human malignancy, providing further evidence for the role of the PI3K pathway in cancer [2]. Many industrial and academic groups paid attention to selective PI3K inhibitors [3] and several of these inhibitors have entered to Phase II clinical trials, such as Pictilisib dimethanesulfonate (GDC-0941, Figure 1). GDC-0941 is the first selective PI3Kα inhibitor developed by Genentech with a half maximal inhibitory concentration (IC_50_) value of 3 nM [4].

**Figure 2:**
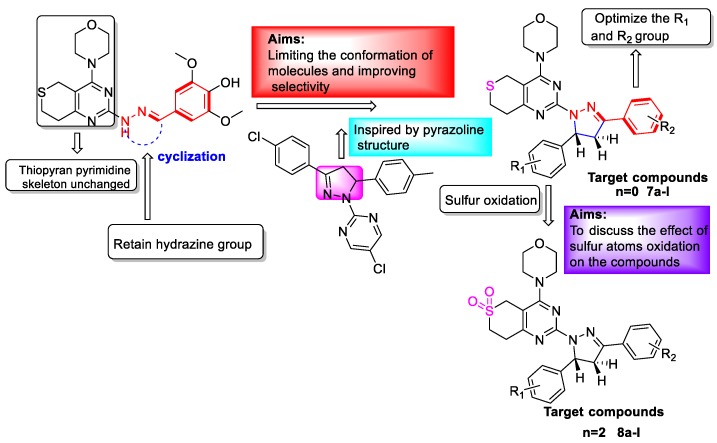
Structures and design strategy for the target compounds (**7a**–**l** and **8a**–**l**).

In our previous study, a series of thiopyrano-pyrimidine compounds containing aromatic hydrazone structures were designed and synthesized, and **Structure-Activity Relationships** (SARs**)** were discussed and summarized. The results showed that these compounds had strong cytotoxicity activity. The activity of the most active compound I (Figure 1) is superior to the lead compound 4-((2-(4,6-dimorpholino-1,3,5-triazin-2-yl)hydrazono)methyl)-2,6-dimethoxyphenol (BMCL-200908069-1, Figure 1). The SARs results of target compounds demonstrated that the construction of thiopyrano-pyrimidine nucleus and the introduction of hydrazine group are beneficial to the cytotoxicity activity of target compounds. However, the activity of these compounds is not as good as GDC-0941, and the inhibitory activity against PI3K kinase is not ideal. This is inconsistent with the expected goal of obtaining good PI3Kα kinase inhibitors [5]. It is necessary to further optimize the previous synthesized compounds. In order to study the effect of changing the structure of the aromatic hydrazone on antitumor activity, we plan to optimize the structure of compound I. As we know, many pyrazoline derivatives have been reported to exhibit various pharmacological activities, such as compounds II and III [6] (Figure 1), and pyrazoline unit was proved to be a good pharmacophore. Therefore, inspired by pyrazoline derivatives, we decided to retain the thiopyrano-pyrimidine nucleus structure, the hydrazine group is incorporated into the ring to construct pyrazoline unit. Finally, we designed and synthesized a series of pyrazole-containing thiopyrano pyrimidine compounds **7a–l**. The different substituents were introduced into the C-4 position of the benzene ring to investigate their effect on the activity. In order to further investigate the effect of oxidation of the sulfur atom on cytotoxicity activity, compounds **8a–l** were prepared via oxidize reaction. As a result, two series of 4-morpholino-7,8-dihydro-5*H*-thiopyrano[4,3-*d*]pyrimidine derivatives (**7a–l** and **8a–l**) bearing pyrazoline moiety were designed and synthesized (Figure 1 and Figure 2).

Herein, we disclosed the synthesis and antitumor activity of all the target compounds against four cancer cell lines human lung cancer cell lines A549, human prostate cancer cell lines PC-3, human breast cancer cell lines MCF-7, and human hepatoma cell lines HepG2 of thiopyrano[4,3-*d*]pyrimidine bearing pyrazoline moiety, as well as the activity against PI3Kα kinase. In addition, we also introduce the docking studies reulsts.

## 2. Results and Discussion

**Scheme 1:**
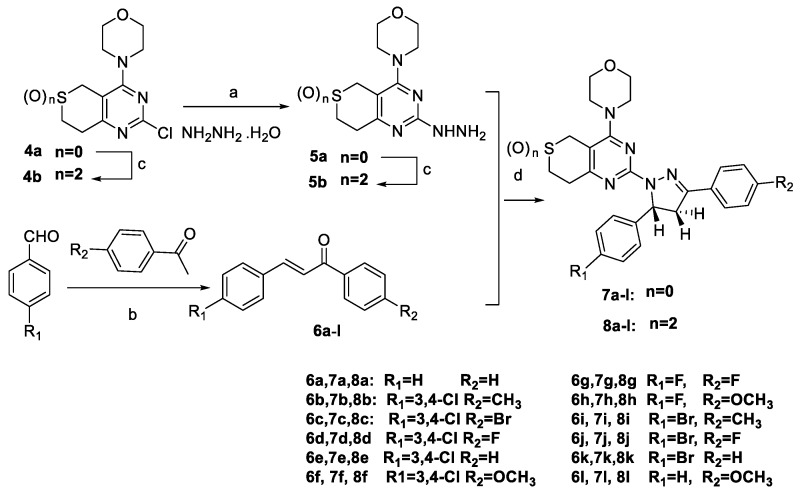
Synthetic route of target compounds. *Reagents and conditions*: (**a**) 80% NH_2_NH_2_·H_2_O, EtOH, 78 °C 1 h; (**b**) 10% NaOH, EtOH, r.t., 24 h; (**c**) Na_2_WO_4_·2H_2_O, 30% H_2_O_2_, 20 °C, 3 h; (d) Glacial acetic acid, H_2_SO_4_; 100 °C.

The synthetic routes to obtain the compounds **7a–l** and **8a–l** are outlined in Scheme 1.

The key intermediates **4a** and **4b** were synthesized from commercially available 3,3′-thiomalo-dimethyl malonate through five steps, which were reported in our previously research [5]. Compounds **4a** and **4b** were each treated with 80% hydrazine monohydrate in refluxing ethanol, generating **5a** and **5b** [7]. Then, **5a** and **5b** were each reacted with chalcone **6a**–**l**, all of which were synthesized according to reported procedures [6] to yield the target compounds (**7a**–**l** and **8a**–**l**). The relative stereochemistry of the target compounds (**7a**–**l**, **8a**–**l**) was confirmed as *R* isomeric according to the method in our previous research [6]. The target compounds **7a**–**l** and **8a**–**l** were established on the basis of ^1^H-NMR. In a typical example, in the ^1^H-NMR spectrum of compound **8d**, the chiral proton of the pyrazoline ring appeared as a doublet of a doublet centered at d 5.63 with a coupling constant *J* = 11.9 Hz and *J* = 5.7 Hz integrating for one proton. The pro-chiral CH_2_ protons of pyrazoline ring appeared as one distinct doublet of a doublet centered at d 3.87 (due to geminal and vicinal coupling and having a coupling constant of 17.7 Hz and 12.4 Hz), and one of the hydrogen of methylene groups is mixed in morpholine.

### 2.1. Biological Evaluation

Taking GDC-0941 as the reference compound, the target compounds (**7a**–**l** and **8a**–**l**) were evaluated for the cytotoxicity against four cancer cell lines A549, PC-3, MCF-7, and HepG2 by 3-(4,5-dimethylthiazolyl-2)-2,5-diphenyltetrazolium bromide (MTT) cell proliferation assay. In order to confirm the types of target compound, selective or mixed-type inhibitors, the selected compound, **8d**, was measured for its PI3Kα kinase inhibitory activity together with reference compounds GDC-0941 and PI103 via the Kinase-Glo^®^ Luminescent time-resolved fluorescence resonance energy transfer (TR-FRET) assay [7]. The results expressed as inhibition rates or IC_50_ values are summarized in Table 1 and Table 2, where the values are the average of at least two independent experiments.

The results of the cytotoxicity assay of the compounds are summarized in Table 1. As expected, we can see that most of the target compounds showed moderate cytotoxicity against the four tested cancer cell lines and most of them showed better activities against PC-3 and HepG2 cancer cell lines than the other two cancer cell lines (A549, MCF-7). Compared with compounds **7a**–**l**, the cytotoxicity activities of compounds **8a**–**l** were decreased or lost. This result shows that the oxidation of sulfur atom was not benefical to the cytotoxicity. However, the cytotoxicity against the four cancer cell lines of compounds **7a**–**l** and **8a**–**l** varied in degree because of the different substituents of the benzene ring at the C-4 position. As seen in Table 1, the electron-withdrawing group-containing compounds (**7c**, **7d**, **7g**, and **7j**) have better antitumor activity than those compounds containing electron-donating groups (**7h**, **7l**). This result indicates that compounds of which the substituents of benzene ring at the C-4 position are electron-withdrawing groups such as substituents (Cl, F, Br) have better activity than that of the compounds containing the electron donating groups (OCH_3_, H). The most promising compound, **8d**, showed moderate cytotoxicity activity to GDC-0941 with the IC_50_ values of 6.02 ± 1.22 μM, 8.91 ± 0.72 μM, 8.39 ± 1.91 μM, and 10.27 ± 0.94 μM. The results show that halogen atom is a strong electron-withdrawing group, and the introduction of halogen atom can affect the drug charge distribution, thereby enhancing the electrical interaction with the receptor.

The activities of the selected compound **8d** as well as the lead compounds against PI3Kα kinase are shown in Table 2. The results showed that compound **8d** exhibited moderate inhibitory rate against PI3Kα kinase, and it indicated that the replacement of aromatic hydrazone with pyrazoline moiety increased the activity against PI3Kα kinase.

### 2.2. Molecular Docking Study

To explore the binding modes of target compounds with the active site of PI3Kα, molecular docking simulation studies were carried out using a SURFLEX-DOCK module [8] of the SYBYL package. Based on the in vitro inhibition results, we selected compound **8d** as the ligand example, and the structures of PI3Kα (Protein Data Bank (PDB) ID code: 4L23 [7]) were selected as the docking models.

**Figure 3:**
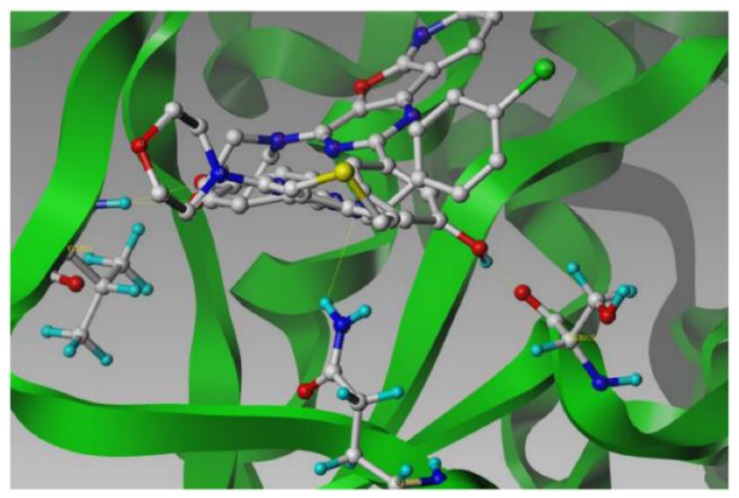
Binding models of Compound **8d** (shown in orange sticks) and native ligand PI103 (shown in blue sticks) with PI3Kα. The proteins are represented by green ribbon. Hydrogen bonds are showed in dashed lines (yellow).

The binding mode of compound **8d** with the active site of PI3Kα molecular is shown in Figure 3. As depicted in Figure 3, the morpholine groups of compound **8d** and PI103 were almost completely overlapped. In the docking model of compound **8d** with PI3Kα, we can see the pyrimidine on the 2-position nitrogen formed one hydrogen bond with residue Asp2357. In addition, a nitrogen atom on a pyrazole structure formed one hydrogen bond with residue Asn2343 and the fluorine atom of the benzene group also formed one hydrogen bond with residue His2340, respectively. The above-mentioned results of SARs analysis and molecular docking study may allow for the rational design of PI3Kα inhibitors.

## 3. Experimental Section

### 3.1. General Information

All melting points were obtained on a Büchi Melting Point B-540 apparatus (Büchi Labortechnik, Flawil, Switzerland) and were uncorrected. NMR spectra were performed using Bruker 400 MHz spectrometers (Bruker Bioscience, Billerica, MA, USA) with TMS as an internal standard. Mass spectra (MS) were taken in electrospray ionization (ESI) mode on Agilent 1100 Liquid chromatography–mass spectrometry (LC-MS) (Agilent, Palo Alto, CA, USA). Thin layer chromatography (TLC) analysis was carried out on silica gel plates GF254 (Qindao Haiyang Chemical, Qingdao, China). All materials were obtained from commercial suppliers and used without purification, unless otherwise specified. Yields were optimized.

### 3.2. Chemistry

#### 3.2.1. General Procedure for Preparation of Compounds **4a**–**b** and **5a**–**b**

Compounds **4a**–**b** and **5a**–**b** were synthesized according to the reported procedures by our research group [5,7].

#### 3.2.2. General Procedure for the Preparation of Compounds **6a**–**l**

Suitably substituted acetophenone (0.1 mol) and substituted aromatic aldehyde (0.1 mol) were dissolved in ethanol (30 mL). To the clear reaction mixture, 10% NaOH was added dropwise. The reaction mixture was agitated and allowed to stand at room temperature for 24 h. The precipitated crystals of **6a**–**l** were collected by filtration, washed, and recrystallized from EtOH.

#### 3.2.3. General Procedure for the Preparation of Target Compounds **7a**–**l** and **8a**–**l**

Substituted chalcone (0.001 mol) and substituted 4-(2-hydrazinyl-7,8-dihydro-5*H*-thiopyrano[4,3-*d*]pyrimidin-4-yl)morpholine (0.001 mol) or 2-hydrazinyl-4-7,8-dihydro-5*H*-thiopyrano[4,3-*d*]pyrimidine 6,6-dioxide (0.001 mol) were dissolved in 20 mL of glacial acetic acid. Catalytic amount of conc. H_2_SO_4_ was added. We then obtained the target compounds, **7a**–**l** and **8a**–**l**, according to the method reported in the literature [6].

*(R)-4-(2-(3,5-Diphenyl-4,5-dihydro-1H-pyrazol-1-yl)-7,8-dihydro-5H-thiopyrano[4,3-d]pyrimidin-4-yl)morpholine* (**7a**). A brown yellow solid. Yield: 70%. m.p. 139–142 °C. ESI-MS *m*/*z*: [M + H]^+^ 457.6; ^1^H-NMR (400 MHz, DMSO) δ 7.76 (d, *J* = 7.9 Hz, 2H, Ar-H), 7.43 (d, *J* = 7.8 Hz, 3H, Ar-H), 7.27 (d, *J* = 7.7 Hz, 2H, Ar-H), 7.20 (d, *J* = 7.6 Hz, 3H, Ar-H), 5.63 (dd, *J* = 11.9, 5.5 Hz, 1H, pyrazoline), 3.87 (dd, *J* = 17.7, 12.3 Hz, 1H, pyrazoline), 3.55 (d, *J* = 14.9 Hz, 3H, thiopyrano and morpholine hydrogen), 3.52–3.43 (m, 3H, thiopyrano and morpholine hydrogen), 3.40 (s, 1H, pyrazoline), 3.10 (dd, *J* = 17.3, 5.4 Hz, 3H, thiopyrano and morpholine hydrogen), 2.99–2.88 (m, 5H, thiopyrano and morpholine hydrogen).^1^^3^C-NMR (100 MHz, DMSO) δ 165.44, 164.41, 155.51, 151.31, 144.43, 132.53, 129.78 129.13(2C), 128.97(2C), 127.34, 126.69(2C), 126.02(2C), 108.86, 66.28(2C), 62.72, 49.14(2C), 42.36, 33.81, 26.19, 26.06. Purity: 98.27% by high performance liquid chromatography (HPLC) (80:20 MeOH/H_2_O).

(*R)-4-(2-(5-(3,4-Dichlorophenyl)-3-(p-tolyl)-4,5-dihydro-1H-pyrazol-1-yl)-7,8-dihydro-5H-thiopyrano[4,3-d]pyrimidin-4-yl)morpholine* (**7b**). A pale yellow solid. Yield: 72%. m.p. 194–196 °C. ESI-MS *m*/*z*: [M + H]^+^ 540.5; ^1^H-NMR (400 MHz, DMSO) δ 7.64 (d, *J* = 7.9 Hz, 2H, Ar-H), 7.56–7.49 (m, 2H, Ar-H ), 7.24 (d, *J* = 7.9 Hz, 2H, Ar-H), 7.17 (d, *J* = 8.3 Hz, 1H, Ar-H), 5.60 (dd, *J* = 12.1, 6.1 Hz, 1H, pyrazoline), 3.82 (dd, *J* = 17.8, 12.2 Hz, 1H, pyrazoline ), 3.51 (dt, *J* = 23.0, 13.2 Hz, 7H, thiopyrano, morpholine and pyrazoline hydrogen), 3.17–3.07 (m, 3H, thiopyrano and morpholine hydrogen), 2.95–2.86 (m, 5H, thiopyrano and morpholine hydrogen), 2.33 (s, 3H, –CH_3_). Purity: 97.50% by HPLC (80:20 MeOH/H_2_O).

(R)-4-(2-(3-(4-Bromophenyl)-5-(3,4-dichlorophenyl)-4,5-dihydro-1H-pyrazol-1-yl)-7,8-dihydro-5H-*thiopyrano[4,3-d]pyrimidin*-4-yl)morpholine (**7c**). A pale yellow solid. Yield: 78%. m.p. 185–186 °C. ESI-MS m/z: [M + H]^+^ 605.38;^1^H-NMR (400 MHz, DMSO) δ 7.68 (d, *J* = 8.5 Hz, 2H, Ar-H), 7.63 (d, *J* = 8.6 Hz, 2H, Ar-H), 7.54 (d, *J* = 8.3 Hz, 2H, Ar-H), 7.18 (d, *J* = 8.1 Hz, 1H, Ar-H), 5.63 (dd, *J* = 12.2, 6.2 Hz, 1H, pyrazoline ), 3.84 (dd, *J* = 17.8, 12.4 Hz, 1H, pyrazoline), 3.67–3.32 (m, 7H ,thiopyrano, morpholine and pyrazoline hydrogen), 3.19–3.05 (m, 3H, thiopyrano and morpholine hydrogen), 2.98–2.87 (m, 5H, thiopyrano and morpholine hydrogen ).^1^^3^C-NMR (100 MHz, DMSO) δ 166.02, 164.52, 162.57, 155.66, 150.31, 145.42, 132.08, 131.63, 131.34, 129.84(2C), 128.829(2C), 126.46, 123.04, 109.43, 66.30(2C), 62.02, 49.22(2C), 41.78, 33.97, 26.22, 26.09. Purity: 98.60% by HPLC (80:20 MeOH/H_2_O).

*(R)-4-(2-(5-(3,4-Dichlorophenyl)-3-(4-fluorophenyl)-4,5-dihydro-1H-pyrazol-1-yl)-7,8-dihydro-5H-thiopyrano[4,3-d]pyrimidin-4-yl)morpholine* (**7d**). A pale yellow solid. Yield: 71%. m.p. 207–211 °C. ESI-MS *m*/*z*: [M + H]^+^ 544.47; ^1^H-NMR (400 MHz, DMSO) δ 7.68 (d, *J* = 8.5 Hz, 2H, Ar-H), 7.63 (d, *J* = 8.6 Hz, 2H, Ar-H), 7.54 (d, *J* = 8.3 Hz, 2H, Ar-H), 7.18 (d, *J* = 8.1 Hz, 1H, Ar-H), 5.63 (dd, *J* = 12.2, 6.2 Hz, 1H, pyrazoline), 3.84 (dd, *J* = 17.8, 12.4 Hz, 1H, pyrazoline), 3.67–3.32 (m, 7H, thiopyrano, morpholine and pyrazoline hydrogen), 3.19–3.05 (m, 3H, thiopyrano and morpholine hydrogen), 2.98–2.87 (m, 5H, thiopyrano and morpholine hydrogen). Purity: 95.31% by HPLC (80:20 MeOH/H _2_O).

*(R)-4-(2-(5-(3,4-Dichlorophenyl)-3-phenyl-4,5-dihydro-1H-pyrazol-1-yl)-7,8-dihydro-5H-thiopyrano[4,3-d]pyrimidin-4-yl)morpholine* (**7e**). A yellow solid. Yield: 73%. m.p. 288–289 °C. ESI-MS *m*/*z*: [M + H]^+^ 556.5; ^1^H-NMR (400 MHz, DMSO) δ 7.75 (d, *J* = 6.7 Hz, 2H, Ar-H), 7.55 (d, *J* = 8.2 Hz, 2H, Ar-H), 7.44 (d, *J* = 7.7 Hz, 3H, Ar-H), 7.18 (d, *J* = 8.3 Hz, 1H, Ar-H ), 5.63 (dd, *J* = 12.1, 6.1 Hz, 1H, pyrazoline), 3.86 (dd, *J* = 17.7, 12.0 Hz, 1H, pyrazoline), 3.63–3.42 (m, 7H, thiopyrano, morpholine and pyrazoline hydrogen), 3.13 (d, *J* = 14.0 Hz, 3H, thiopyrano and morpholine hydrogen), 3.00–2.88 (m, 5H, thiopyrano and morpholine hydrogen). Purity: 97.40% by HPLC (80:20 MeOH/H_2_O).

*(R)-4-(2-(5-(3,4-Dichlorophenyl)-3-(4-methoxyphenyl)-4,5-dihydro-1H-pyrazol-1-yl)-7,8-dihydro-5H-thiopyrano[4,3-d]pyrimidin-4-yl)morpholine* (**7f**). A yellow solid. Yield: 75%. m.p. 197–209 °C. ESI-MS *m*/*z*: [M + H]^+^ 464.1; ^1^H-NMR (400 MHz, DMSO) δ 7.81 (d, *J* = 8.7 Hz, 2H, Ar-H), 7.60–7.55 (m, 2H, Ar-H), 7.22 (dd, *J* = 8.4, 1.8 Hz, 1H, Ar-H), 7.04 (d, *J* = 8.8 Hz, 2H, Ar-H), 5.65 (dd, *J* = 11.8, 6.1 Hz, 1H, pyrazoline), 3.93 (dd, *J* = 18.1, 11.9 Hz, 1H, pyrazoline), 3.81 (s, 3H,–OCH_3_), 3.56 (dd, *J* = 3.6 Hz, 4H, morpholine), 3.42 (d, *J* = 14.1, 6.9 Hz, 3H, thiopyrano and pyrazoline hydrogen), 3.26–2.83 (m, 8H, thiopyrano and morpholine hydrogen). Purity: 97.04% by HPLC (80:20 MeOH/H_2_O).

*(R)-4-(2-(3,5-Bis(4-fluorophenyl)-4,5-dihydro-1H-pyrazol-1-yl)-7,8-dihydro-5H-thiopyrano[4,3-d]pyrimidin-4-yl)morpholine* (**7g**). A pale yellow solid. Yield: 70%. m.p. 144–146°C.. ESI-MS *m*/*z*: [M + H]^+^ 493.58; ^1^H-NMR (400 MHz, DMSO) δ 8.08 (d, *J* = 2.8 Hz, 2H, Ar-H), 7.60–7.50 (m, 4H, Ar-H), 7.39 (s, 2H, Ar-H), 5.63 (dd, *J* = 12.1, 6.1 Hz, 1H, pyrazoline), 3.86 (dd, *J* = 17.7, 12.0 Hz, 1H, pyrazoline), 3.63–3.42 (m, 7H, thiopyrano, morpholine and pyrazoline hydrogen), 3.13 (d, *J* = 14.0 Hz, 3H, thiopyrano and morpholine hydrogen), 3.00–2.88 (m, 5H, thiopyrano and morpholine hydrogen). ^13^C-NMR (100 MHz, DMSO) δ 165.87, 164.55, 162.75, 161.92, 160.33, 155.77, 150.21, 140.53, 129.16, 128.90, 128.83, 128.11, 128.04, 116.25, 116.03, 115.78, 115.57, 109.03, 66.28(2C), 62.10(2C), 49.17, 42.32, 26.25, 26.07. Purity: 95.20% by HPLC (80:20 MeOH/H_2_O).

*(R)-4-(2-(5-(4-Fluorophenyl)-3-(4-methoxyphenyl)-4,5-dihydro-1H-pyrazol-1-yl)-7,8-dihydro-5H-thiopyrano[4,3-d]pyrimidin-4-yl)morpholine* (**7h**). A yellow solid. Yield: 68%. m.p. 116–118 °C. ESI-MS *m*/*z*: [M + H]^+^ 505.6; ^1^H-NMR (400 MHz, DMSO) δ 8.06 (d, *J* = 8.3 Hz, 2H, Ar-H), 7.58–7.52 (m, 2H, Ar-H), 7.41 (t, *J* = 8.6 Hz, 2H,Ar-H), 7.30 (d, *J* = 8.4 Hz, 2H, Ar-H), 5.92 (dd, *J* = 11.7, 5.6 Hz, 1H, pyrazoline), 4.18 (dd, *J* = 17.9, 12.1 Hz, 1H, pyrazoline), 4.08 (s, 3H, –OCH_3_), 3.84–3.72 (m, 7H, thiopyrano, morpholine and pyrazoline hydrogen), 3.50 (dd, *J* = 26.7, 8.6 Hz, 4H, thiopyrano and morpholine hydrogen), 3.22 (dd, *J* = 29.1, 5.3 Hz, 4H, thiopyrano and morpholine hydrogen). Purity: 95.80% by HPLC (80:20 MeOH/H_2_O).

*(R)-4-(2-(5-(4-Bromophenyl)-3-(p-tolyl)-4,5-dihydro-1H-pyrazol-1-yl)-7,8-dihydro-5H-thiopyrano[4,3-d]pyrimidin-4-yl)morpholine* (**7i**). A yellow solid. Yield: 77%. m.p. 142–145 °C. ESI-MS *m*/*z*: [M + H]^+^ 475.59; ^1^H-NMR (400 MHz, DMSO) δ 7.64 (d, *J* = 7.2 Hz, 2H, Ar-H), 7.47 (d, *J* = 7.2 Hz, 2H, Ar-H), 7.24 (d, *J* = 7.4 Hz, 2H, Ar-H), 7.15 (d, *J* = 7.2 Hz, 2H, Ar-H), 5.63 (dd, *J* = 12.1, 6.1 Hz, 1H, pyrazoline), 3.86 (dd, *J* = 17.7, 12.0 Hz, 1H, pyrazoline), 3.63–3.42 (m, 7H, thiopyrano, morpholine and pyrazoline hydrogen), 3.13 (d, *J* = 14.0 Hz, 3H, thiopyrano and morpholine hydrogen), 3.00–2.88 (m, 5H, thiopyrano and morpholine hydrogen), 2.32 (s, 3H, CH_3_).^1^^3^C-NMR (100 MHz, DMSO) δ 165.91, 164.53, 155.78, 151.17, 144.00, 139.50, 131.84(2C), 129.72(2C), 128.41(2C), 126.67(2C), 120.18, 108.94, 66.29(2C), 62.24, 116.03, 49.19(2C),42.16, 33.97, 26.25, 26.05, 21.45. Purity: 95.54% by HPLC (80:20 MeOH/H_2_O).

*(R)-4-(2-(5-(4-Bromophenyl)-3-(4-fluorophenyl)-4,5-dihydro-1H-pyrazol-1-yl)-7,8-dihydro-5H-thiopyrano[4,3-d]pyrimidin-4-yl)morpholine* (**7j**). A pale yellow solid. Yield: 71%. m.p. 220–221 °C. ESI-MS *m*/*z*: [M + H]^+^ 550.5; ^1^H-NMR (400 MHz, DMSO) δ 7.79 (s, 2H, Ar-H), 7.30–7.20 (m, 4H, Ar-H), 7.11 (d, *J* = 7.9 Hz, 2H, Ar-H), 5.68–5.57 (m, 1H, pyrazoline), 3.83 (dd, *J* = 16.9, 12.6 Hz, 1H, pyrazoline), 3.64–3.41 (m, 7H, thiopyrano, morpholine and pyrazoline hydrogen), 3.08 (d, *J* = 12.2 Hz, 3H, thiopyrano and morpholine hydrogen), 2.95–2.85 (m, 5H, thiopyrano and morpholine hydrogen). Purity: 97.34% by HPLC (80:20 MeOH/H_2_O).

*(R)-4-(2-(5-(4-Bromophenyl)-3-phenyl-4,5-dihydro-1H-pyrazol-1-yl)-7,8-dihydro-5H-thiopyrano[4,3-d]pyrimidin-4-yl)morpholine* (**7k**). A yellow solid. Yield: 69%. m.p. 167–168 °C. ESI-MS *m*/*z*: [M + H]^+^ 536.49; ^1^H-NMR (400 MHz, DMSO) δ 7.79–7.71 (m, 2H, Ar-H), 7.48–7.37 (m, 3H, Ar-H), 7.24 (dd, *J* = 8.5, 5.6 Hz, 2H, Ar-H), 7.10 (t, *J* = 8.8 Hz, 2H, Ar-H), 5.65 (dd, *J* = 12.1, 5.6 Hz, 1H, pyrazoline), 3.85 (dd, *J* = 17.7, 12.2 Hz, 1H, pyrazoline), 3.67–3.37 (m, 7H, thiopyrano, morpholine and pyrazoline hydrogen), 3.16–3.05 (m, 3H, thiopyrano and morpholine hydrogen), 2.97–2.87 (m, 5H, thiopyrano and morpholine hydrogen). Purity: 96.03% by HPLC (80:20 MeOH/H_2_O).

*(R)-4-(2-(3-(4-Methoxyphenyl)-5-phenyl-4,5-dihydro-1H-pyrazol-1-yl)-7,8-dihydro-5H-thiopyrano[4,3-d]pyrimidin-4-yl)morpholine* (**7l**). A pale yellow solid. Yield: 67%. m.p. >300 °C. ESI-MS *m*/*z*: [M + H]^+^ 487.6; ^1^H -NMR (400 MHz, DMSO) δ 7.72 (d, *J* = 8.4 Hz, 2H, Ar-H), 7.28 (d, *J* = 7.0 Hz, 2H, Ar-H), 7.21 (d, *J* = 7.6 Hz, 3H, Ar-H), 7.01 (d, *J* = 8.5 Hz, 2H, Ar-H), 5.63 (s, 1H, pyrazoline), 3.86 (d, *J* = 17.3 Hz, 1H, pyrazoline), 3.81 (s, 3H, –OCH_3_), 3.56 (dd, *J* = 3.6 Hz, 4H, morpholine), 3.42 (d, *J* = 14.1, 6.9 Hz, 3H, thiopyrano and pyrazoline hydrogen), 3.26–2.83 (m, 8H, thiopyrano and morpholine hydrogen). Purity: 98.86% by HPLC (80:20 MeOH/H_2_O).

*(R)-2-(3,5-Diphenyl-4,5-dihydro-1H-pyrazol-1-yl)-4-morpholino-7,8-dihydro-5H-thiopyrano[4,3-d]pyrimidine 6,6-dioxide* (**8a**). A pale yellow solid. Yield: 70%. m.p. 219–220 °C. ESI-MS *m*/*z*: [M + H]^+^ 489.5; ^1^H-NMR (400 MHz, DMSO) δ 7.90 (d, *J* = 6.3 Hz, 2H, Ar-H), 7.40 (d, *J* = 7.3 Hz, 4H, Ar-H), 7.28 (d, *J* = 3.2 Hz, 2H, Ar-H), 7.23 (d, *J* = 7.6 Hz, 2H, Ar-H ), 5.60 (dd, *J* = 11.9, 6.2 Hz, 1H, pyrazoline),4.12 (dd, *J* = 39.3, 15.5 Hz, 2H, thiopyrano), 3.87 (dd, *J* = 17.7, 12.4 Hz, 1H, pyrazoline), 3.56 (s, 2H, morpholine), 3.51 (s, 2H, morpholine), 3.43 (s, 2H, thiopyrano), 3.25–3.15 (m, 3H, morpholine and pyrazoline hydrogen), 3.08 (s, 2H, morpholine), 2.97 (s, 2H, thiopyrano). Purity: 97.60% by HPLC (80:20 MeOH/H_2_O).

*(R)-2-(5-(3,4-Dichlorophenyl)-3-(p-tolyl)-4,5-dihydro-1H-pyrazol-1-yl)-4-morpholino-7,8-dihydro-5H-thiopyrano[4,3-d]pyrimidine* *6,6-dioxide* (**8b**). A yellow solid. Yield: 63%. m.p. 225–226 °C. ESI-MS *m*/*z*: [M + H]^+^ 572.50; ^1^H-NMR (400 MHz, DMSO) δ 7.65 (d, *J* = 8.0 Hz, 2H,Ar-H), 7.54 (d, *J* = 8.8 Hz, 2H, Ar-H), 7.25 (d, *J* = 8.0 Hz, 2H, Ar-H), 7.16 (d, *J* = 8.2 Hz, 1H, Ar-H), 5.62 (dd, *J* = 12.1, 5.9 Hz, 1H, pyrazoline), 4.16 (d, *J* = 15.6 Hz, 1H, thiopyrano), 4.06 (d, *J* = 15.5 Hz, 1H, thiopyrano), 3.85 (dd, *J* = 17.9, 12.3 Hz, 1H, pyrazoline), 3.56 (s, 2H, morpholine), 3.49 (d, *J* = 9.0 Hz, 2H, morpholine), 3.43 (s, 2H, thiopyrano), 3.16 (dd, *J* = 17.9, 5.9 Hz, 3H, morpholine and pyrazoline hydrogen), 3.12–3.05 (m, 2H, morpholine), 2.97 (s, 2H, thiopyrano), 2.33 (s, 3H, –CH_3_). Purity: 99.45% by HPLC (80:20 MeOH/H_2_O).

*(R)-2-(3-(4-Bromophenyl)-5-(3,4-dichlorophenyl)-4,5-dihydro-1H-pyrazol-1-yl)-4-morpholino-7,8-dihydro-5H-thiopyrano[4,3-d]pyrimidine* *6,6-dioxide* (**8c**). A yellow solid. Yield: 59%. m.p. 162–163 °C. ESI-MS *m*/*z*: [M + H]^+^ 637.37; ^1^H-NMR (400 MHz, DMSO) δ 7.68 (d, *J* = 8.4 Hz, 2H, Ar-H), 7.62 (d, *J* = 8.3 Hz, 2H, Ar-H), 7.54 (d, *J* = 6.9 Hz, 2H,Ar-H), 7.17 (d, *J* = 8.4 Hz, 1H, Ar-H), 5.62 (dd, *J* = 12.1, 6.0 Hz, 1H, pyrazoline), 4.16 (d, *J* = 15.6 Hz, 1H, thiopyrano), 4.08 (d, *J* = 15.6 Hz, 1H,thiopyrano), 3.86 (dd, *J* = 17.8, 12.3 Hz, 1H, pyrazoline), 3.56 (d, *J* = 5.3 Hz, 2H,morpholine), 3.53–3.46 (m, 2H, morpholine), 3.43 (s, 2H, thiopyrano), 3.24–3.14 (m, 3H, morpholine and pyrazoline hydrogen), 3.09 (d, *J* = 11.8 Hz, 2H,morpholine), 2.97 (s, 2H, thiopyrano). Purity: 96.89% by HPLC (80:20 MeOH/H_2_O).

*(R)-2-(5-(3,4-Dichlorophenyl)-3-(4-fluorophenyl)-4,5-dihydro-1H-pyrazol-1-yl)-4-morpholino-7,8-dihydro-5H-thiopyrano[4,3-d]pyrimidine 6,6-dioxide* (**8d**). A yellow solid. Yield: 61%. m.p. 150–153 °C. ESI-MS m/z: [M + H]^+^ 576.47; ^1^H-NMR (400 MHz, DMSO) δ 7.85–7.77 (m, 2H, Ar-H), 7.55 (d, *J* = 6.6 Hz, 2H, Ar-H), 7.28 (t, *J* = 8.4 Hz, 2H, Ar-H), 7.18 (d, *J* = 8.1 Hz, 1H, Ar-H), 5.63 (dd, *J* = 11.9, 5.7 Hz, 1H, pyrazoline), 4.12 (dd, *J* = 39.3, 15.5 Hz, 2H, thiopyrano), 3.87 (dd, *J* = 17.7, 12.4 Hz, 1H, pyrazoline), 3.56 (s, 2H, morpholine), 3.51 (s, 2H, morpholine), 3.43 (s, 2H, thiopyrano), 3.25–3.15 (m, 3H, morpholine and pyrazoline hydrogen), 3.08 (s, 2H, morpholine), 2.97 (s, 2H, thiopyrano). ^1^^3^C-NMR (100 MHz, DMSO) δ 165.77, 164.58, 162.55, 162.12, 156.38, 151.39, 145.22, 131.42, 129.91, 129.16, 129.16(2C), 129.08, 128.80, 126.38(2C), 116.31, 116.10, 102.61, 66.17(2C), 61.83, 49.04(2C), 47.03, 32.48. Purity: 95.14% by HPLC (80:20 MeOH/H_2_O).

*(R)-2-(5-(3,4-Dichlorophenyl)-3-phenyl-4,5-dihydro-1H-pyrazol-1-yl)-4-morpholino-7,8-dihydro-5H-thiopyrano[4,3-d]pyrimidine* *6,6-dioxide* (**8e**). A yellow solid. Yield: 57%. m.p. 245–249 °C. ESI-MS *m*/*z*: [M + H]^+^ 588.5; ^1^H-NMR(400 MHz, DMSO) δ 7.85–7.77 (m, 2H, Ar-H), 7.55 (d, *J* = 6.6 Hz, 2H, Ar-H), 7.28 (t, *J* = 8.4 Hz, 2H,Ar-H), 7.18 (d, *J* = 8.1 Hz, 2H, Ar-H), 5.63 (dd, *J* = 11.9, 5.7 Hz, 1H, pyrazoline ), 4.12 (dd, *J* = 39.3, 15.5 Hz, 2H, thiopyrano), 3.87 (dd, *J* = 17.7,12.4 Hz, 1H, pyrazoline), 3.56 (s, 2H, morpholine), 3.51 (s, 2H, morpholine), 3.43 (s, 2H, thiopyrano), 3.25–3.15 (m, 3H, morpholine and pyrazoline hydrogen), 3.08 (s, 2H, morpholine), 2.97 (s, 2H, thiopyrano). Purity: 96.53% by HPLC (80:20 MeOH/H_2_O).

*(R)-2-(5-(3,4-Dichlorophenyl)-3-(4-methoxyphenyl)-4,5-dihydro-1H-pyrazol-1-yl)-4-morpholino-7,8-dihydro-5H-thiopyrano[4,3-d]pyrimidine* *6,6-dioxide* (**8f**). A pale yellow solid. Yield: 55%. m.p. 203–205 °C. ESI-MS *m*/*z*: [M + H]^+^ 558.4; ^1^H-NMR (400 MHz, DMSO) δ 7.70 (d, *J* = 8.0 Hz, 2H, Ar-H), 7.53 (d, *J* = 10.9 Hz, 2H, Ar-H), 7.17 (d, *J* = 8.0 Hz, 1H, Ar-H), 6.99 (d, *J* = 8.0 Hz, 2H, Ar-H), 5.59 (dd, *J* = 11.4, 5.3 Hz, 1H, pyrazoline), 4.11 (dd, *J* = 35.3, 15.4 Hz, 2H, thiopyrano), 3.89–3.82 (m, 1H, pyrazoline), 3.79 (s, 3H, –OCH_3_), 3.54 (d, *J* = 17.8 Hz, 4H, morpholine), 3.42 (s, 2H, thiopyrano), 3.19 (d, *J* = 5.6 Hz, 2H, morpholine), 3.15–3.05 (m, 3H, morpholine and pyrazoline hydrogen), 2.97 (s, 2H, thiopyrano). Purity: 98.66% by HPLC (80:20 MeOH/H_2_O).

*(R)-2-(3,5-bis(4-Fluorophenyl)-4,5-dihydro-1H-pyrazol-1-yl)-4-morpholino-7,8-dihydro-5H-thiopyrano[4,3-d]pyrimidine* *6,6-dioxide* (**8g**). A yellow solid. Yield: 53%. m.p. 246–249 °C. ESI-MS *m*/*z*: [M + H]^+^ 525.5; ^1^H-NMR (400 MHz, DMSO) δ 7.71 (d, *J* = 8.0 Hz, 2H, Ar-H), 7.23 (s, 2H, Ar-H), 7.11 (t, *J* = 8.1 Hz, 2H, Ar-H), 7.00 (d, *J* = 8.0 Hz, 2H, Ar-H), 5.62 (d, *J* = 7.0 Hz, 1H, pyrazoline), 4.12 (dd, *J* = 39.3, 15.5 Hz, 2H, thiopyrano), 3.87 (dd, *J* = 17.7, 12.4 Hz, 1H, pyrazoline), 3.56 (s, 2H, morpholine), 3.51 (s, 2H, morpholine), 3.43 (s, 2H, thiopyrano), 3.25–3.15 (m, 3H, morpholine and pyrazoline hydrogen), 3.08 (s, 2H, morpholine), 2.97 (s, 2H, thiopyrano). Purity: 98.38% by HPLC (80:20 MeOH/H_2_O).

*(R)-2-(5-(4-Fluorophenyl)-3-(4-methoxyphenyl)-4,5-dihydro-1H-pyrazol-1-yl)-4-morpholino-7,8-dihydro-5H-thiopyrano[4,3-d]pyrimidine 6,6-dioxide* (**8h**). A yellow solid. Yield: 50%. m.p. 140–142 °C. ESI-MS m/z: [M + H]^+^ 537.6; ^1^H-NMR (400 MHz, DMSO) δ 7.71 (d, *J* = 8.0 Hz, 2H,Ar-H), 7.23 (s, 2H, Ar-H), 7.11 (t, *J* = 8.1 Hz, 2H,Ar-H), 7.00 (d, *J* = 8.0 Hz, 2H, Ar-H), 5.62 (d, *J* = 7.0 Hz, 1H, pyrazolin), 4.16 (d, *J* = 15.4 Hz, 1H, thiopyrano), 4.04 (d, *J* = 15.5 Hz, 1H, thiopyrano), 3.85 (d, *J* = 17.6 Hz, 1H, pyrazolin), 3.79 (s, 3H, –OCH_3_), 3.57 (s, 2H, morpholine), 3.51 (s, 2H, morpholine), 3.43 (s, 2H, thiopyrano), 3.18 (s, 2H, morpholine), 3.08 (d, *J* = 17.9 Hz, 3H, morpholine and pyrazoline hydrogen), 2.95 (s, 2H, thiopyrano). ^1^^3^C-NMR (100 MHz, DMSO) δ 165.82, 162.76, 162.43, 160.91, 160.35, 156.45, 152.00, 140.51, 128.43, 128.06, 127.99, 124.91, 115.84, 115.63(2C), 114.65(2C), 102.07, 66.16(2C), 61.81, 55.79, 49.29, 49.05, 47.11, 42.47, 32.46. Purity: 99.04% by HPLC (80:20 MeOH/H_2_O).

*(R)-2-(5-(4-Bromophenyl)-3-(p-tolyl)-4,5-dihydro-1H-pyrazol-1-yl)-4-morpholino-7,8-dihydro-5H-thiopyrano[4,3-d]pyrimidine* *6,6-dioxide* (**8i**). A yellow solid. Yield: 48%. m.p. 282–283 °C. ESI-MS *m*/*z*: [M + H]^+^ 507.5; ^1^H-NMR (400 MHz, DMSO) δ 7.65 (d, *J* = 7.9 Hz, 2H, Ar-H), 7.47 (d, *J* = 8.2 Hz, 2H, Ar-H), 7.24 (d, *J* = 7.9 Hz, 2H, Ar-H), 7.16 (d, *J* = 8.3 Hz, 2H, Ar-H), 5.60 (dd, *J* = 11.9, 5.4 Hz, 1H, pyrazolin), 4.17 (d, *J* = 15.5 Hz, 1H, thiopyrano), 4.05 (d, *J* = 15.5 Hz, 1H, thiopyrano), 3.84 (dd, *J* = 17.7, 12.2 Hz, 1H, pyrazolin), 3.56 (s, 2H, morpholine), 3.51 (d, *J* = 3.4 Hz, 2H, morpholine), 3.42 (d, *J* = 5.2 Hz, 2H, thiopyrano), 3.19 (d, *J* = 5.1 Hz, 2H, morpholine), 3.11–3.03 (m, 3H, morpholine and pyrazoline hydrogen), 2.98 (s, 2H, thiopyrano), 2.32 (s, 3H, –CH_3_). Purity: 98.63% by HPLC (80:20 MeOH/H_2_O).

*(R)-2-(5-(4-Bromophenyl)-3-(4-fluorophenyl)-4,5-dihydro-1H-pyrazol-1-yl)-4-morpholino-7,8-dihydro-5H-thiopyrano[4,3-d]pyrimidine* *6,6-dioxide* (**8j**). A yellow solid. Yield: 49%. m.p. 158–163 °C. ESI-MS *m*/*z*: [M + H]^+^ 582.5; ^1^H-NMR (400 MHz, DMSO) δ 7.71 (d, *J* = 8.0 Hz, 2H, Ar-H), 7.23 (s, 2H, Ar-H), 7.11 (t, *J* = 8.1 Hz, 2H, Ar-H), 7.00 (d, *J* = 8.0 Hz, 2H, Ar-H), 5.62 (d, *J* = 7.0 Hz, 1H, pyrazoline),4.12 (dd, *J* = 39.3, 15.5 Hz, 2H, thiopyrano), 3.87 (dd, *J* = 17.7, 12.4 Hz, 1H, pyrazoline), 3.56 (s, 2H, morpholine), 3.51 (s, 2H, morpholine), 3.43 (s, 2H, thiopyrano), 3.25–3.15 (m, 3H, morpholine and pyrazoline hydrogen), 3.08 (s, 2H, morpholine), 2.97 (s, 2H, thiopyrano). Purity: 98.37% by HPLC (80:20 MeOH/H_2_O).

*(R)-2-(5-(4-Bromophenyl)-3-phenyl-4,5-dihydro-1H-pyrazol-1-yl)-4-morpholino-7,8-dihydro-5H-thiopyrano[4,3-d]pyrimidine 6,6-dioxide* (**8k**). A pale yellow solid. Yield: 52%. m.p. 163–167 °C. ESI-MS *m*/*z*: [M + H]^+^ 568.4; ^1^H-NMR (400 MHz, DMSO) δ 7.76 (d, *J* = 6.8 Hz, 2H, Ar-H ), 7.45 (dd, *J* = 21.0, 6.9 Hz, 5H, Ar-H), 7.17 (d, *J* = 7.4 Hz, 2H, Ar-H), 5.62 (dd, *J* = 11.7, 5.1 Hz, 1H, pyrazoline), 4.17 (d, *J* = 15.4 Hz, 1H,thiopyrano), 4.05 (d, *J* = 15.5 Hz, 1H, thiopyrano), 3.87 (dd, *J* = 17.4, 12.4 Hz, 1H, pyrazoline), 3.57 (s, 2H, morpholine), 3.49 (d, *J* = 11.4 Hz, 4H, morpholine and thiopyrano hydrogen), 3.22–3.12 (m, 3H, morpholine and pyrazoline hydrogen), 3.11–3.04 (m, 2H, morpholine), 2.97 (s, 2H, thiopyrano).^1^^3^C-NMR (100 MHz, DMSO) δ 165.75, 162.41, 156.34, 152.17, 143.60, 132.24, 131.93(2C), 130.04, 129.17(2C), 128.39(2C), 126.82(2C), 120.35, 102.38, 66.16(2C), 62.09, 49.29(2C), 49.01, 47.03, 42.17, 32.44. Purity: 96.29% by HPLC (80:20 MeOH/H_2_O).

*(R)-2-(3-(4-Methoxyphenyl)-5-phenyl-4,5-dihydro-1H-pyrazol-1-yl)-4-morpholino-7,8-dihydro-5H-thiopyrano[4,3-d]pyrimidine* *6,6-dioxide* (**8l**). A pale yellow solid. Yield: 50%. m.p. 109–114 °C. ESI-MS *m*/*z*: [M + H]^+^ 519.6; ^1^H-NMR (400 MHz, DMSO) δ 7.71 (d, *J* = 8.7 Hz, 2H, Ar-H), 7.31–7.24 (m, 2H, Ar-H ), 7.19 (d, *J* = 6.9 Hz, 3H, Ar-H), 6.99 (d, *J* = 8.8 Hz, 2H, Ar-H), 5.59 (dd, *J* = 11.9, 5.4 Hz, 1H, pyrazoline), 4.15 (d, *J* = 15.5 Hz, 1H, thiopyrano), 4.02 (d, *J* = 15.4 Hz, 1H, thiopyrano), 3.89–3.82 (m, 1H, pyrazoline), 3.78 (s, 3H, –OCH_3_), 3.53 (d, *J* = 5.8 Hz, 2H, morpholine), 3.49–3.38 (m, 4H, morpholine and thiopyrano hydrogen), 3.22–3.15 (m, 2H, morpholine), 3.12–3.00 (m, 3H, morpholine and pyrazoline hydrogen), 2.93 (s, 2H, thiopyrano). Purity: 98.22% by HPLC (80:20 MeOH/H_2_O).

### 3.3. Cytotoxicity Assay In Vitro

The in vitro cytotoxic activities of Compounds **7a**–**l** and **8a**–**l** were evaluated with A549, PC-3, HepG2, and MCF-7 cell lines by the standard MTT assay, with GDC-0941 as a positive control. The cancer cell lines were cultured in minimum essential medium (MEM) supplement with 10% fetal bovine serum (FBS). Approximately, 4 × 10^3^ cells, suspended in MEM medium, were plated onto each well of a 96-well plate and incubated in 5% CO_2_ at 37 °C for 24 h. The test compounds at the indicated final concentrations were added to the culture medium, and cell cultures continued for 72 h. Fresh MTT was added to each well at a terminal concentration of 5 µg/mL and incubated with cells at 37 °C for 4 h. The formazan crystals were dissolved in 100 µL of DMSO in each well, and the absorbency at 492 nm (for absorbance of MTT formazan) and 630 nm (for the reference wavelength) was measured with an enzyme linked immunosorbent assay (ELISA) reader (MR-96A Mindray Elisa Microplate Reader, Guangzhou, China). All compounds were tested three times in each of the cell lines. The results expressed as inhibition rates or IC_50_ were the averages of two determinations and calculated using the Bacus Laboratories Inc. Slide Scanner (Bliss) software (the Bacus Laboratories Inc. Slide Scanner (BLISS) system, Lombard, IL, USA) [7].

### 3.4. PI3Kα Kinase Assay

The selected compound **8d** was tested for its activity against PI3Kα using a Kinase-Glo^®^Luminescent Kinase Assay (Promega, Madison, WI, USA), with GDC-0941 and PI103 as positive controls. The kinase reaction occurred in a 384-well black plate. Each well was loaded with 50 µL of test items (in 90% DMSO) and 5 µL of reaction buffer containing 10 µg/mL PI substrate (l-α-phosphatidylinositol; Avanti Polar Lipids (Avanti Polar Lipids, Inc., Alabaster, AL, USA); prepared in 3% octyl-glucoside), and the PI3Kα protein (10 nM) was then added to it. The reaction was started by the addition of 5 µL of 1 µM Adenosine triphosphate (ATP) prepared in the reaction buffer and incubated for 60 min for p110α. It was terminated by the addition of 10 µL of Kinase-Glo buffer. The plates were then read in a Synergy 2 reader (BioTek, Winooski, VT, USA) for luminescence detection. This assay was repeated twice [7].

### 3.5. Docking Studies

For docking purposes, we prepared the receptor proteins PDB ID code: 4L23 (PI3Kα). The three-dimensional structure of the PI3Kα was obtained from the Research Collaboratory for Structural Bioinformatics (RCSB) Protein Data Bank. We built a small organic molecule set (compound **8d**) and used the Gasteiger–Huckel method to optimize the molecular force field and structure. Hydrogen atoms were added to the structure allowing for appropriate ionization at physiological pH. First, ligand substructure was extracted, water and excess structure were then removed, and hydrogens and fix sidechain amides were finally added. The protonated state of several important residues, such as Asp2357, Asn2343, and His2340 were adjusted using SYBYL6.9.1 (Tripos, St. Louis, MO, USA) in favor of forming a reasonable hydrogen bond with the ligand. Molecular docking analysis was carried out by the SURFLEX-DOCK module of the SYBYL 6.9.1 package to explore the binding model for the active site of PI3Kα with its ligand. All atoms located within the range of 5.0 Å from any atom of the cofactor were selected into the active site, and the corresponding amino acid residue was, therefore, involved into the active site if only one of its atoms was selected. Other default parameters were adopted in the SURFLEX-DOCK calculations. All calculations were performed on a Silicon Graphics workstation (package version 6.9.1 on silicon graphics origin300 workstation with IRIX 6.5 operating system, San Francisco, CA, USA). Lastly, docking results and the optimized molecular docking model with the receptor proteins were obtained [7].

## 4. Conclusions

In summary, two series of 4-morpholino-7,8-dihydro-5*H*-thiopyrano[4,3-*d*]pyrimidine derivatives bearing pyrazoline scaffolds were designed, synthesized, and evaluated for cytotoxicity activity against four cancer cell lines and the PI3Kα kinase in vitro*.* The pharmacological results indicated that most of the compounds showed moderate cytotoxicity activity against the four cancer cell lines. In particular, the results indicated that the most promising compound **8d** showed excellent cytotoxicity against four cancer cell lines with IC_50_ values of 6.02–10.27 μM. In addition, compound **8d** was found to have a moderate inhibitory activity toward PI3Kα enzyme. The initial SARs and docking studies with PI3Kα molecular showed that the morpholine group and the pyrazoline scaffold were necessary for these compounds to possess potent cytotoxicity activities. What’s more, various types of substituents impacted differently on the activity of the two series of compounds. The compounds in which the substituents of benzene ring at the C-4 position are electron-withdrawing groups such as substituents (Cl, F, Br) have better activity than the compounds containing the electron donating groups (OCH_3_, H). Further studies will be carried out in the near future.

## Figures and Tables

**Table 1:**
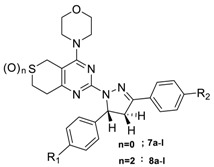
Structures and cytotoxicity of Compounds **7a**–**l** and **8a**–**l**.

Compd.	n	R_1_	R_2_	IC_50_ (μM) ^a^			
				A549	PC-3	HepG2	MCF-7
**7a**	0	H	H	21.68 ± 0.76	28.8 ± 2.40	20.34 ± 1.15	21.74 ± 0.78
**7b**	0	3,4-diCl	CH_3_	24.17 ± 0.74	14.09 ± 3.45	27.81 ± 0.99	42.65 ± 1.75
**7c**	0	3,4-diCl	Br	13.02 ± 0.42	12.879 ± 1.56	15.36 ± 0.03	22.98 ± 0.19
**7d**	0	3,4-diCl	F	6.02 ± 1.22	8.91 ± 0.72	8.39 ± 1.91	10.27 ± 0.94
**7e**	0	3,4-diCl	H	NA	37.76 ± 3.64	NA	35.89 ± 0.71
**7f**	0	3,4-diCl	OCH_3_	NA	NA	NA	NA
**7g**	0	F	F	16.92 ± 0.66	15.15 ± 1.54	20.27 ± 0.37	26.35 ± 0.37
**7h**	0	F	OCH_3_	26.72 ± 0.88	29.82 ± 0.89	20.47 ± 0.67	30.26 ± 0.88
**7i**	0	Br	CH_3_	16.41 ± 0.43	25.43 ± 0.74	NA	27 .29 ± 1.85
**7j**	0	Br	F	19.25 ± 1.25	14.75 ± 1.02	23.83 ± 0.55	28.25 ± 0.14
**7k**	0	Br	H	31.19 ± 0.59	17.83 ± 0.55	32.73 ± 0.36	35.68 ± 0.74
**7l**	0	H	OCH_3_	36.38 ± 0.62	28.25 ± 0.14	NA	NA
**8a**	2	H	H	27.58 ± 0.85	NA	32.73 ± 0.39	41.25 ± 1.25
**8b**	2	3,4-diCl	CH_3_	35.65 ± 1.47	17.80 ± 1.18	NA	42.46 ± 0.03
**8c**	2	3,4-diCl	Br	27.80 ± 1.18	37.58 ± 0.22	0.06 ± 0.01	43.86 ± 0.15
**8d**	2	3,4-diCl	F	23.84 ± 0.67	19.06 ± 0.87	30.67 ± 0.09	NA
**8e**	2	3,4-diCl	H	NA	NA	NA	35.31 ± 0.49
**8f**	2	3,4-diCl	OCH_3_	NA	38.65 ± 0.18	35.01 ± 0.18	NA
**8g**	2	F	F	29.08 ± 0.49	21.84 ± 0.67	36.01 ± 0.18	35.32 ± 0.39
**8h**	2	F	OCH_3_	34.97 ± 0.74	26.55 ± 0.33	32.91 ± 2.82	33.87 ± 0.45
**8i**	2	Br	CH_3_	28.08 ± 0.89	31.89 ± 0.25	24.68 ± 0.74	NA
**8j**	2	Br	F	29.66 ± 0.32	31.86 ± 0.92	27.76 ± 0.64	46.63 ± 0.89
**8k**	2	Br	H	34.99 ± 0.47	33.52 ± 1.67	35.49 ± 0.33	32.61 ± 0.49
**8l**	2	H	OCH_3_	NA	37.38 ± 0.23	NA	NA
**GDC-0941 ^b^**				6.99 ± 0.21	0.20 ± 0.08	0.07 ± 0.03	4.30 ± 0.84

^a^ The half maximal inhibitory concentration (IC_50_) values are an average of two separate determinations; ^b^ Used as a positive control; NA: No Activity.

**Table 2:** Structures and cytotoxicity of Compounds **7a**–**l** and **8a**–**l**.

Compound	IC_50_^a^ (µM)
	PI3Kα^c^
**8d**	8.38 ± 0.21
**GDC-0941** **^b^**	0.003
**PI-103** **^b^**	0.075 ± 0.018

^a^ The IC_50_ values are an average of two separate determinations; ^b^ Used as a positive control; ^c^ PI3Kα, Phosphatidylinositol 3-kinase α.

## References

[1] Crabbe T., Welham M.J., Ward S.G. (2007). The PI3K inhibitor arsenal: Choose your weapon!. Trends Biochem. Sci..

[2] Wymann M.P., Zvelebil M., Laffargue M. (2003). Phosphoinositide 3-kinase signaling—Which way to target?. Trends Pharmacol. Sci..

[3] Heffron T.P., Wei B., Olivero A., Staben S.T., Tsui V., Do S., Dotson J., Folkes A.J., Goldsmith P., Goldsmith R. (2011). Rational design of phosphoinositide 3-kinase α inhibitors that exhibit selectivity over the phosphoinositide 3-kinase β isoform. J. Med. Chem..

[4] Sutherlin D.P., Sampath D., Berry M., Castanedo G., Chang Z., Chuckowree I., Dotson J., Folkes A., Friedman L., Goldsmith R. (2010). Discovery of (Thienopyrimidin-2-yl)aminopyrimidines as Potent, Selective, and Orally Available Pan-PI3-Kinase and Dual Pan-PI3-Kinase/mTOR Inhibitors for the Treatment of Cancer. J. Med. Chem..

[5] Sun C., Chen C., Xu S., Wang J., Zhu Y., Kong D., Tao H., Jin M., Zheng P., Zhu W. (2016). Synthesis and anticancer activity of novel 4-morphlino-7,8-dihydro-5*H*-thiopyrano[3,4-*d*]pyrimidine derivatives bearing chromone moiety. Bioorg. Med. Chem..

[6] Adhikari A., Kallurayaa B., Sujitha K.V., Gouthamchandrab K., Jairamc R., Mahmoodb R., Sankollid R. (2012). Synthesis, characterization and pharmacological study of 4,5-dihydropyrazolines carrying pyrimidine moiety. Eur. J. Med. Chem..

[7] Zhu W., Sun C., Xu S., Wu C., Wu J., Xu M., Zheng P. (2014). Design, synthesis, anticancer activity and docking studies of novel 4-morpholino-7,8-dihydro-5*H*-thiopyrano[4,3-*d*]pyrimidine derivatives as mTOR inhibitors. Bioorg. Med. Chem..

[8] Jain A.N. (2007). Surflex-Dock 2.1: Robust performance from ligand energetic modeling, ring flexibility, and knowledge-based search. J. Comput. Aided Mol. Des..

